# The Hospital. Nursing Section

**Published:** 1902-08-16

**Authors:** 


### The Hospital.

### Nursing Section.

Contributions for this Section of "The Hospital" should be addressed to the Editor, "The Hospital" Nursing Section, 28 & 29 Southampton Street, Strand, London, W.C.

No. 829.—Vol. XXXII. *SATURDAY, AUGUST* 16, 1902.

### Notes on News from the Nursing World.

### THE KING'S NURSES AT THE CORONATION.

The King did not forget his two nurses on Coronation Day. They were assigned seats in Westminster Abbey and had the gratification of witnessing the great historic ceremony. They were attired in hospital uniform, and the appreciation by the public of their services was manifested in a striking manner. As they drove along the route in the Royal carriage, drawn by a pair of chestnut horses, they were repeatedly cheered. On their arrival at Westminster, an old lady from Darlington, who saw the Coronation procession of Queen Victoria, and was in her place on the stand at St. Margaret's Church at 8 o'clock, observed with glee, "It's just like the King to do that."

### NURSES AND THE CORONATION PROCESSION.

Although, happily, the services of nurses were not much in request on Coronation Day, a wise provision had been made in case of need. Under the auspices of the Army Medical Corps, there were four stations in the vicinity of Westminster Abbey. At the station in Cloister Green, Superintendent-Sister L. J. Mackay, R.R.C., and Sister J. A. Evans, were on duty; at the station in Poets' Corner Green, Superintendent-Sister E. Ryan, R.R.C., and Sister A. W. Walkinshaw; at the station in North Abbey Green, Sisters L. M. Steward and A. F. Hislop; and at the station near the west entrance of the Abbey, Superintendent-Sister S. F. Hart, R.R.C., and Sister A. E. Tait. In another column a Sister gives an interesting description of her duty on Coronation Day. In connection with the St. John's Ambulance Brigade upwards of a hundred nurses were also on duty at different points of the route, prepared to render assistance. The nursing staff at the Westminster Hospital, to which some Indian soldiers injured by a frightened horse after the Coronation were conveyed, had the privilege of surveying the scene outside the Abbey from a row of seats erected by the managers of the institution. On the Parliament Gardens stand were many sisters, Colonials and others, returned from the front, some who only arrived in London the evening before obtaining seats through the kind offices of Lady Dudley. Sisters' wardrobes are apt to run down on active service, and the regulation bonnet is almost universally discarded for the more serviceable sailor hat, panama, or light felt, and among those on the stand there were very few in the red hooded cloak and bonnet of the A.N.S.R. Indeed, that many of them were sisters at all could hardly be judged save by the wearing of the Reserve badge or the South African ribbon or medal. It was amusing to note that discrepancies of uniform notwithstanding, the Sisters were, as a rule, recognised by Tommy Atkins, and from time to time one heard, "There's the medal," "That's a Sister," "Look, there's one of them." Once, passing a party of soldiers on their way to take up their position on the route, the order "Eyes right for the Sisters" was given and loyally responded to; and when the King had entered the Abbey many were the interested glances at those perambulating in the crowd to stretch themselves, after the long confinement on the stands, who could be made out to have returned from the front. In the crowd in Parliament Street were many nurses who evidently had been unable to secure seats, but who, nevertheless, may have had an excellent view of the procession, for the police will always, if possible, help a nurse to get a good view of anything of interest going forward.

### THE ALEXANDRA NURSES.

To the Queen, who presided at the annual meeting of the Soldiers and Sailors' Families Association on the eve of the Coronation, no portion of the report read by Colonel Gildea was, perhaps, more interesting than the account of the progress of the nursing branch of the organisation. The work, in which fourteen nurses are now employed, continues to be appreciated and in many garrisons has become a necessity. We do not doubt that, as the report says, the more it becomes known the more it will increase. Special recognition is given by the council to the services of Nurse Brown at Pietermaritzburg, of whom it is stated that during the last three years her work "has been most valuable, not only in attending to the wives and families, but also in helping with the outdoor patients of the Army Medical Department during the war." This is a great personal compliment to Miss Brown, as well as an evidence of the excellent manner in which the skill and energy of the Alexandra nurses can be turned to account in extraordinary circumstances. The cost of the branch last year to the Association amounted to £900, but about an equal sum was raised locally at the stations where the nurses are engaged.

### RETIREMENT OF LORD RAGLAN.

It will be noted that the Ministerial changes consequent upon the accession of Mr. Arthur Balfour to the Premiership include the substitution of Lord Hardwicke for Lord Raglan as Under Secretary of State for War. Lord Hardwicke is an able and energetic young peer. But nurses were interested in Lord Raglan because, as might have been expected from the bearer of the name, he has on more than one occasion gone out of his way to show that he has followed the development of the nursing movement with attention, and it was reflected with satisfaction that the grandson of the Commander-in-Chief of the British forces in the Crimea was one of the friends of nurses in the department. Lord Raglan's appointment to the post of Lieutenant-Governor of the Isle of Man has since been announced.

### NURSES FROM SOUTH AFRICA.

The members of Queen Alexandra's Imperial Military Nursing Service who have arrived from South Africa since the publication of our last issue are, on the *Dunolly Castle*, Sisters N. S. Searle and R. Gwyer; on the *Pembroke Castle*, Sisters M. McCreery, M. Careswell, and M. L. Harris; on the *Alnwick Castle*, Sisters C. M. C. Rogers and J. Lawson; on the *Corinthian*, Sisters A. M. Poulter, F. Epton, Rogers, and Cleghorn, duty on voyage; on the *Kildonan Castle*, Sisters C. Heaton, M. E. Andrews, L. Ainsworth, J. Booth, E. D. Reynolds, B. E. Hutchison, and M. A. Macdonald, duty on voyage; on the *Goorkha*, Sisters A. E. French, M. E. Powell, F. E. Filkin, A. Marton, A. A. Pallot, and S. C. Schlieman. Owing to the reduction of the establishment none of these nurses will be required to return. The *Saxon*, which is due on Saturday, has on board Sisters L. Hand and R. M. Browse. The *Simla*, due August 24th, has on board Sisters M. W. Pughe, M. A. Richards, G. E. Larner, A. G. Hayhurst, S. A. Boneham, G. E. S. Saunder, K. A. G. Chew, M. G. Gilmore, and D. A. Gray. The *Orissa*, due August 21st, has on board Sisters A. D. Cameron, A. A. Hill, N. R. Mackintosh, and A. W. C. Higgs. The *Dilwara*, also due August 21st, has on board Sisters J. C. S. Spence, E. N. Newton, L. Parsons, and E. M. Pethick. The *Gascon*, due August 24th, has on board Sisters G. McFarlane, M. G. Denton, A. Matherson, S. J. Coldwell, E. Grey, and E. R. Tait. The *Moravian*, due August 26th, has on board Sisters F. E. Beedie and E. F. Ridgley. The *Nubia*, due August 28th, has on board Sisters H. M. Shaw, A. R. Denton, S. M. Paterson, A. Finnemore, Carr, A. B. Cullum, E. Despard, J. Paget, F. Sykes, K. X. Phillips, M. Pedlar, and L. M. Green.

### THE UP-COUNTRY NURSING ASSOCIATION.

The honorary secretary sends us, with the seventh report of the Up-Country Nursing Association for Europeans in India, of which Queen Alexandra is patron, and to which she has just contributed a donation of £50, a copy of the rules for the engagement and employment of nurses. These rules were only adopted at a meeting of the working committee on the 28th of last month. It may be useful to mention that applicants for appointments should exceed 25 years of age, should have had "at least three years'" training in a recognised general hospital, and that preference will be given to those who are competent to practise as midwives, or have had experience as maternity nurses. The salary offered is 75 rupees or £5 a month, and the allowances are: a second-class saloon passage to India; £10 for railway journey and incidental travelling expenses; £20 for an outfit allowance, with which it is required that the prescribed uniform shall be purchased; board, washing, attendance, and medical attendance. The ordinary term of service is five years, but it may be shortened to three, or extended by the local committee of the Association. A return passage free to England is only, however, granted to nurses who have served for five or more years, except in case of serious illness. A nurse who resigns without the consent of the local committee before the close of her agreed term of service, is required to refund the money paid for her passage from London to Bombay, and, unless she has completed her two years' service, the outfit allowance. Short intervals of leave or rest may be granted on full pay from time to time at the discretion of the local committee. During 1901 two nurses were sent out by the Association, one to the North-Western Provinces and the other to the Punjab. Since 1894 twenty nurses have gone to India under its auspices.

### MOUNT VERNON CONSUMPTION HOSPITAL.

The nurses of the Mount Vernon Consumption Hospital, Hampstead, have passed a highly successful and difficult examination on the nursing of consumption. The papers, we are informed, were all well thought out, showed an unusual amount of interest and a close attention to the lectures on the part of the candidates.

### CROYDON UNION INFIRMARY.

The state of affairs at Croydon Union Infirmary continues to be unsatisfactory. We are constantly receiving letters which indicate that, as a training school, under present circumstances it is not an institution to be eagerly sought, if only because none of the nurses know what will happen next. It seems, too, that the nurses instead of being appointed or at least selected by the matron, are now required to apply to either the medical superintendent or the guardians. So long as this is the case there is sure to be unpleasantness, and we think that the time has arrived when the authorities of the Local Government Board should take decisive action. Otherwise, Croydon Infirmary threatens to become a by-word in the nursing world.

### THE GLASGOW NURSES' GUILD AND CLUB.

It will be remembered that we gave an account in December of the opening of a Nurses' Guild and Club at Glasgow. We are glad to hear that there are now no fewer than 170 members, a roll-call which proves that the efforts of the committee of ladies who collected sufficient money to give the venture a start have already been highly appreciated. The conditions of membership are certainly not difficult to fulfil. In addition to an entrance fee of half-a-crown, there is an annual subscription of the same amount. The advantages of joining are many. Members can have a bedroom at a small charge per week and their meals on moderate terms. They may also introduce two friends as guests. The rooms of the club being in the centre of the city, nurses engaged in private work find them very convenient for rest, change, and reading of papers, as well as for refreshments. The object of the guild is to arrange Sunday services, communion at suitable hours for nurses, and to help any individual nurse who may desire guidance. During the winter the ladies' committee hope to get up social evenings and entertainments; and for the information of our readers who may like to know more about the matter, we add that full particulars can be obtained from Mrs. E. J. Ritchie, the Hon. Sec., 6 Westminster Gardens, Hillhead, Glasgow.

### THE LATE MISS FLORENCE MEYRICK.

The death of Miss Florence Meyrick, who since 1892 was lady superintendent of the Nursing Hostel in Beaumont Street, London, will be widely regretted. Miss Meyrick who was trained at St. Mary's Hospital, Paddington, and the Hospital for Sick Children, in Great Ormond Street. From 1874 to 1890 she was lady superintendent of the Hospital for Gentlewomen in Harley Street. An intensely religious woman, she had always the courage of her convictions, and she was deservedly respected for her consistency and high principles.

### HALIFAX INFIRMARY.

On Friday the Royal Halifax Infirmary was the scene of a pleasing little ceremony in commemoration of the Coronation of King Edward VII. and Queen Alexandra. Each member of the nursing staff was presented with a beautifully bound copy of King Edward VII. Coronation Prayer-book by the president of the institution, Mr. Ed. Huntriss. The ward, house, and laundrymaids were presented with Coronation brooches of a pretty design in blue enamel and silver, and about £10 in new half-crowns were distributed amongst the patients. On Saturday tobacco for the male and cakes for the female patients were provided by the house surgeons.

### THE MATRONSHIP OF THE HOWARD DE WALDEN HOME.

The House Committee of the Howard de Walden Home in Langham Street are advertising for a matron. They offer a trained nurse, competent bookkeeper, and accountant a salary of £60 per annum, with board, lodging, and laundry. The successful applicant will need an abundant supply of tact, and the power to hold her own in the event of complications arising.

### FOREIGN NURSING INSTITUTIONS.

English nurses who propose to become attached to a nursing institution abroad should make the most careful inquiries beforehand. Some very unpleasant experiences seem to have fallen to the share of a couple of ladies who, taking, we are afraid, a good many statements on trust, joined an organisation in France on the understanding that it was an English institution, under English management, and conducted in the manner of ordinary institutions in this country. As it was intimated on one of the circulars issued by the management that the training of the nurses was carried "on the principles of the noted Florence Nightingale," there was at least some excuse for their confidence. But, for the rest, it appears that the institution was very un-English. Lack of decent food, the crowding of three or four nurses into one small bedroom, absence of fire in severe weather, and the admission of cases which in England are taken in isolated hospitals, are among the drawbacks which, we are informed, they suffered. They will not have suffered in vain if the recital of their reasonable complaints puts others on their guard against the devices of speculative foreigners who, knowing how much the services of English-trained nurses are appreciated on the Continent, attempt to make money out of them without any concern for either their health, feelings, or comfort.

### DIET AT ST. GERMANS INFIRMARY.

It is extremely important that the dietary of nurses employed as officers of the Local Government Board should not differ according to the workhouse infirmaries in which they are engaged. There have been complaints of the meals at St. Germans Infirmary, and a new nurse has expressed her surprise that the food supplied is not so good as it was in two other workhouse infirmaries of which she has lately had experience. The master of the workhouse at St. Germans has since made inquiries and has ascertained that the food of the nurses and of the master and matron there is not so good as that of the domestic servants at Marylebone and other workhouses. This should certainly be remedied, or St. Germans Workhouse will get a bad name, and efficient nurses will fight shy of it. Moreover, there should be no practical difficulty, and as the question has been referred to the House Committee of the Guardians it may be concluded that a speedy improvement will be effected. Otherwise, the Local Government Board should be asked to intervene.

### ANOTHER PLUCKY NURSE.

Last week we recorded the case of a London nurse who gave chase to a suspected thief, and today we note that of a Pontefract nurse who saved a little boy from drowning. As Miss Holman was passing the pond outside the park gates at Pontefract she saw the child fall into the water, which is about 2½ feet in depth. He disappeared in the mud at the bottom, and the nurse, who promptly jumped into the water after him, had considerable difficulty in discovering his whereabouts. However, she eventually found him, brought him to land, and carried him into an adjacent cottage, where artificial respiration was successfully applied.

### A TRIBUTE TO LADY COLLECTORS.

At the fourth annual meeting of the Colchester Nursing Association, Dr. Fenn presented the report, which showed that the total number of cases received during the year ended June 21 was 148, and the number of visits paid 5,123. The balance-sheet, which Dr. Fenn also submitted, indicated a balance in hand of £6 8s. 5d., and he said that the satisfactory financial position of the association was largely due to the strenuous exertions of the lady collectors and the careful supervision exercised by the hon. secretaries. The only adverse comment came from Dr. Macgregor, who drew attention to the fact that subscribers did not pay with the regularity which was their duty. No doubt the lady collectors will see to this in future. Having put the association on a paying basis, they will take care that there is no retrogression.

### COVENTRY GUARDIANS AND THE NURSING INSTITUTION.

The different views of different boards of guardians as to the value of the work of district nurses are constantly being illustrated. At Coventry the Guardians have just decided to increase their subscription to the Nursing Institution from 12 to 20 guineas. Bearing in mind the size of the town and the very considerable proportion of sick poor who need nursing, 20 guineas is a small sum. Yet there were members of the Coventry Board who objected to it, although they must be perfectly well aware of the fact that many of the visits of nurses during the year are paid to persons in receipt of out-door relief, who otherwise would claim provision in case of illness at the hands of the Guardians.

### Lectures to Nurses on Medical Nursing.

By Eveline A. Cargill, M.D.Brux., L.R.C.P.&S.Ed.

### LECTURE IV.—DISEASES OF THE HEART AND CIRCULATION.

There are many different forms of heart disease, but when we say a person has "heart disease" we generally mean that one (or more) of the valves of the heart are affected. Valvular disease is almost invariably confined to the left side of the heart, the right side being rarely affected. On the left side the mitral valve is the most commonly attacked, the aortic less commonly. Inflammation of the mitral valve may occur in childhood in an acute form, usually in the course of a severe illness, as rheumatic or scarlet fever; and its effects are rarely cured, though they may be latent for years; or it may occur in adult life in a more chronic form. The effect of inflammation is to thicken and stiffen the valve and to prevent it from working properly. It may not open sufficiently wide—*stenosis*, causing obstruction in the flow of blood, or it may not close perfectly—*incompetence*, causing regurgitation of the blood, or both these effects may be present at once—*double mitral disease*.

Failure to close properly is the most common effect, and it will be best to describe shortly the series of changes in the circulation due to this affection of the mitral valve, though the symptoms in all forms of disease of this valve are very similar. When the left ventricle contracts, instead of all the blood being forced into the aorta, some flows back or regurgitates through the imperfectly-closed mitral valve. To overcome this difficulty the ventricle becomes thicker and stronger, or hypertrophies, and works more energetically, and as long as it can keep the circulation going, we say "compensation" has taken place, *i.e.*, the weakness or incompetence of the valve is compensated by the extra power of the ventricle. After a time, which may be short or long, the ventricle can increase no more, and then it begins to dilate, the walls yield a little instead of becoming stronger, and now we say compensation is failing, because the heart can no longer keep the circulation going. This is the time of great and increasing danger in heart disease; when the strength of the heart is not sufficient to keep the blood moving on, and it stagnates in the veins and capillaries. Then the liquid part exudes through the vessel walls causing œdema and later general dropsy. The œdema of heart disease is first seen in the feet and ankles, and gradually extends up the legs. It also occurs in the lungs, causing breathlessness and cough, and later bronchitis; in the liver, causing indigestion and jaundice; in the kidney, causing scanty urine and preventing excretion of waste products, and in the abdomen, producing ascites. The stagnation of blood in the veins gives a bright red or purplish tint to the lips, cheeks and ears, and this when associated with a slightly yellowish skin produces an appearance characteristic of mitral disease. The effect of the failing circulation in the brain is seen in drowsiness and restlessness. Death takes place from heart failure or from some complication of lungs, kidneys, etc., and is generally slow.

Disease of the aortic valve is often caused by severe muscular strain or prolonged exertion, and is the form of heart disease most often found in active vigorous men. It is marked by pallor of the face and lips and attacks of faintness, and sudden death is not uncommon, or it may end like mitral disease owing to the mitral valve yielding as the heart dilates, and then we see all the long train of symptoms mentioned above which make death from advanced heart disease one of very severe and often prolonged suffering.

Perhaps the most frequent cause of sudden death is fatty degeneration of the heart. Persons suffering from this disease are mostly elderly, from 50 years old and upwards, but are not necessarily fat people. They have a sallow, waxy skin, are apt to be breathless on slight exertion, and subject to attacks of fainting which become more frequent as time goes on. The disease is often not detected during life, as the symptoms are not marked, and a person suffering from it may not even have consulted a doctor, when he dies suddenly, and then the heart is found to be pale and soft, and the muscular fibres to be largely replaced by fat. It is in this disease that the heart may "break"—the patient attempts some extra exertion, and the weakened heart ruptures—causing immediate death.

Pain is not a prominent feature in heart disease; in 99 cases out of 100 when a patient complains of pain in the region of the heart, it is caused by dyspepsia, and the same is true of palpitations; anæmia, dyspepsia, and "nerves" cause more cardiac discomfort than heart disease. The most painful diseases connected with the heart are pericarditis or inflammation of the membrane surrounding the heart, which occurs in the course of some acute illness such as rheumatic fever or pneumonia, and is very painful. It is more common in children, and as it is sometimes determined by chill, the nurse should be very careful not to expose the chest more than is absolutely necessary for the doctor's examination in a child acutely ill. Great care must also be exercised in changing poultices, fomentations, etc., in such cases.

Another painful disease is angina pectoris, which is a spasm of the heart causing sudden violent and agonising pain in which a patient sometimes dies. Any remedies to be of use must be administered instantly and the nurse must be absolutely certain of the method of giving the medicine and the quantity required that there may be no hesitation or delay in cutting short the attack.

Death by heart disease may come slowly, as in mitral valve disease from stagnation of blood in the veins leading literally to the choking of the whole system, or suddenly as in fatty degeneration of the heart or disease of the aortic valve, by syncope or sudden heart failure.

Syncope or faintness is always due to a weakened action of the heart, and this may be caused by emotion such as fright or joy, or it may be caused by a weakened condition of the heart itself, as in fatty degeneration, or it may be caused by hæmorrhage. Hæmorrhage or bleeding is escape of blood from a blood-vessel either by injury or disease of the vessel wall. If bleeding takes place into the lungs it is coughed up and called "hæmoptysis," if into the stomach it is vomited and called "hæmatemesis," if into the brain it causes apoplexy, and here the danger is from the injury done to the brain substance and not from the amount of blood lost which is generally inconsiderable; or the bleeding may be into an internal organ or cavity such as the abdomen, when it is called "internal hæmorrhage." The symptoms of internal hæmorrhage are those of collapse, and resemble a very slow process of fainting, ending in heart failure. A patient with internal hæmorrhage shows great and increasing pallor of the lips and skin with cold perspiration, restlessness, and an increasingly quick, weak, and soft pulse, respirations are shallow and sighing, and he soon becomes unconscious. The faintness is really curative, as it slows the circulation and gives a chance for the blood at the mouth of the torn vessel to clot. The nurse must keep the patient absolutely quiet with head low, and warmth to the surface, and no stimulants until the doctor can be summoned. The condition is one of extreme danger and no time must be lost in procuring medical assistance.

### The Queen and the Imperial Yeomanry Hospitals.

### PRESENTATION OF MEDALS TO NURSES.

On Monday afternoon a most interesting ceremony took place in the beautiful grounds of Devonshire House. The occasion was the presentation of medals by the Queen to members of the staffs of the Imperial Yeomanry Hospital who had served in South Africa, namely, 30 officers, 250 orderlies and dressers, 25 nurses, and 10 wardmaids.

### The Scene.

All those who were to be honoured by the Queen were marshalled on the lawn, around which a rope was placed so as to separate them from the spectators, a couple of hundred in number, who sat round outside the cord. In one corner of the garden the band of the Scots Guards were stationed, and played at intervals, whilst the King's Company of the Grenadier Guards—surely some of their tallest men—were posted at intervals to keep it clear. The scene was a particularly pretty one. The terrace, which is immediately behind the house, is reached directly through some of the sitting-rooms, and is raised some feet above the rest of the garden. It was carpeted with Oriental rugs, and in the centre was a large umbrella of pale blue and lemon. Under this the Queen was to stand to perform the ceremony of the afternoon, and due preparations had evidently been made. Beside the round table, and in front of the ring of golden armchairs, piled one on the top of another, were half a dozen drawers each containing little boxes with a medal and a ribbon. On the lawn below was a varied throng, constantly changing like a kaleidoscope as they obeyed the word of command and moved to their appointed places. There were doctors in frock coats and high hats, others in summer clothes and straw hats, soldiers in khaki, soldiers in dark uniforms with military caps, whilst here and there the men of the National Fire Brigade Union Ambulance Corps stood out distinct from all the others by reason of their gleaming golden helmets. The nurses, though not numerous, had managed to represent many contrasts of costume. Two were in light summer frocks; others, afraid of the bleakness in the air—although the almanack said that it was summer—had put on their cloaks as well as their bonnets. Some, greatly daring, wore nothing over their pale cotton or dark-blue material dresses except a white apron with a snowy cap on their head, with, here and there, such protection as is offered by the scarlet cape of the Army Nursing Service. On the terrace, amongst the ladies of the committee, were several nurses also of the Imperial Yeomanry, who already possessed their medals, having received them from the King at Marlborough House last July.

### The Topic of the Hour.

As might have been expected, the universal subject of conversation whilst waiting for the Queen was the Coronation, and it transpired that the King's nurses were not the only two who were fortunate enough to obtain seats in the Abbey. One of two Sisters wearing the Army uniform was in the Peers' Gallery, and spoke in terms of enthusiastic admiration of the striking scene which met their eyes when the Queen's crown had been placed upon her head and all the peeresses had followed her example. The only drawback to their pleasure on Saturday was their state of famine. A nurse-friend had taken a nice little packet of sandwiches with her, and her companion had in consequence thought herself in luck, but the possessor of the welcome store was also the possessor of a kind heart, and when she saw the hungry looks of some of the people near her she made a general distribution, with the result that two little sandwiches were all that fell to each Sister's share.

### The Arrival of the Queen.

As the speaker added that her first meal that day had been at 6 A.M., the second at 5 P.M., a distant sound of cheering broke upon the quiet air, and in a moment the Queen appeared in the doorway leaning upon the arm of the Duke of Devonshire. She looked gracious and sweet in a gown made princess fashion of the palest mauve, with a mauve and white toque, and as she passed along the terrace she stopped to speak and shake hands with many of the guests. Amongst others was a little girl of eight or nine summers daintily attired in white and pink, and the kindly greeting of the Queen was duly acknowledged by the child with a delighted smile and the regulation curtsey. Lord Kitchener, seeming very big and burly beside Lord Roberts, bent low over the Royal hand extended to him, and as the Queen passed on, found many others desirous of speaking to him, amongst others the Prince of Wales and his two sisters. There were no speeches, and as soon as Her Majesty had addressed a few words to most of those in her immediate vicinity, the business of the afternoon commenced.

### The Presentation.

The officers filed first up the incline from the lawn, and then came the nurses, whose names are as follows:—Miss K. B. Brereton, night superintendent of the base hospital; Miss W. E. Cheeseman, who acted as housekeeper; Miss E. C. Cheetham, Miss G. Digwood, Miss M. E. Ireland, Miss B. Lancaster, Miss A. MacLeod, Miss H. M. O'Connor, Miss D. Pryde, Miss M. E. Rowell, Miss E. K. Sharp, Miss E. C. Smith, Miss F. J. Smith, Miss B. J. Talbot, Miss E. Tucker, Miss E. F. Uppleby, Miss D. West, Miss L. Whiley, and Miss Young. Each nurse curtsied as she stopped in front of Her Majesty, and again before she passed on after receipt of her medal. To several of the Sisters the Queen said a word or two, and then came the wardmaids and the dressers and orderlies. It was amusing to note how as some of the men left the Royal presence they caught sight of the Sisters who, though they had returned to England some time before, had evidently not been forgotten. Salutes and bows and smiles sufficed as acknowledgment in most cases, but one young fellow was evidently so pleased to see Sister again that heedless of curious eyes he left the beaten path and shook her heartily by the hand.

### After the Ceremony.

At last the final medal was given, and the Queen turned to enter the house for tea. But this move was not speedily accomplished, for there were still many whose good work in the cause Her Majesty wished to acknowledge, and she remained on the terrace some little time after the ceremony was over. After the Royal visitors had departed the general company found much to say to each other, and the photographers were busy with their cameras, Lord Kitchener, of course, being amongst the snap-shotted generals. One of the pictures should prove particularly interesting, and tend to dispel the curious idea that he is a woman-hater, for, when it was taken, he was chatting in an animated manner with a group of young ladies, and laughing heartily at what they were saying.

### The Army Nursing Service Reserve and the South African War.

In the very bulky report of the Central British Red Cross Committee on Voluntary Organisations in aid of the sick and wounded during the South African war, issued under the auspices of the War Office, the work of the Army Nursing Service Reserve is summed up. As this is the official record we give it in full.

The work of the Army Nursing Service Reserve in connection with the voluntary aid resources of the war was confined to the selection and outfit of nursing sisters for duty not only in the hospitals in South Africa, but also in the larger military hospitals at home whose establishment of nursing sisters of the Regular Army Nursing Service had become depleted. This reserve, originally initiated by H.R.H. Princess Christian of Schleswig Holstein (Princess Helena of Great Britain), was established under definite regulations and under the official recognition of the Secretary of State for War in 1897. The qualifications for enrolment in it are similar to the qualifications required for admission into the Army Nursing Service.

### Uniform and Outfit.

The conditions and remuneration for service when on duty in military hospitals has been regulated by the War Office, but supplementary grants have been made to the Army Nursing Service Reserve out of Red Cross funds. Thus the cost of uniform and outfit of a nursing sister of the Reserve amounts to £9 5s. 7d., if the nursing sister proceeded direct to South Africa; to £8 4s. 1d. if she joined a station at home during the winter months; and to £7 16s. 1d. if she joined for home duty during the summer months. There was also an additional expense of 10s. 6d., the cost of badge of membership. Of these sums the Government paid the following:—£8 5s. for each outfit for South Africa; £6 for the winter months' home outfit; and £5 5s. for the summer months' home outfit; the balance of the cost, namely, £1 0s. 7d. for South Africa, £2 4s. 1d. for the winter months at home, and £2 11s. 1d. for the summer months at home, and the cost of the badges, have been provided out of the Red Cross Funds.

### The Roll-Call.

At the commencement of the war on October 9th, 1899, the number of nursing sisters on the list of the Army Nursing Service Reserve was 101. Between that date and May 10th, 1901, 839 were added. The number of applications for enrolment was, up to October 9th, 1899, 141, and between that date and May 10th, 1901, 1,153, or a total of 1,294. The proportion of nursing sisters who applied for enrolment, but had to be rejected in consequence of their not coming up to the standard of qualifications required, was 27 per cent.

### The Red Cross Committee.

It was very desirable that the rules of the Army Nursing Service Reserve should be supported by the Red Cross Committee. In time of war the applications that pour in to the medical service for employment as nurses at the front are numerous and varied. Women of high social position, women with qualifications for nursing, and women with no qualifications, all feel that they have a special mission in connection with the sick and wounded soldiers, and had the rules of the Army Nursing Service Reserve not been rigidly adhered to in the selection of nurses for employment in military hospitals in South Africa, the difficulties of administering the medical service of the war would have been greatly increased.

### The "Princess of Wales's Nurses."

But in order to permit of a desirable amount of elasticity, the Committee of the Army Nursing Service Reserve invariably took into consideration applications made for the special enrolment on their books of nursing sisters belonging to other recognised Nursing Associations and Hospitals, or selected by committees or donors of the private hospitals that were sent out. Thus Her Royal Highness the Princess of Wales (Queen Alexandra), the Honorary President of the Red Cross Committee, intimated her desire on the 19th December, 1899, to select and send 12 nursing sisters to South Africa, to be called the "Princess of Wales's Nurses." The Red Cross Committee at once recognised the value of nursing sisters selected in this manner, and the Committee of the Army Nursing Service Reserve, through the Red Cross Committee, undertook to place upon the roll of the Army Nursing Service Reserve any nursing sisters so selected, and to have them sent to South Africa without delay, provided they otherwise fulfilled the conditions for enrolment. Again, on 18th November, 1899, Lord Justice FitzGibbon, Chairman of the City of Dublin Nursing Institution, wrote to the Central British Red Cross Committee expressing the desire of many of the nurses of the institution to volunteer for service in South Africa, and Her Royal Highness, Princess Christian, President of the Army Nursing Service Reserve, at once arranged a special meeting of the Reserve Committee with a view to selecting for enrolment those nurses of the City of Dublin Nursing Institution who possessed the necessary qualifications. On December 7th, 1899, the Chairman submitted the documents of five selected nurses who were enrolled in the Reserve and sent to South Africa.

### The Local Appointments.

Some difficulty was experienced at one time in connection with the appointment locally in South Africa of nurses, who were in the country, to do duty with military hospitals. Some of these were not so qualified as the nurses of the Army Nursing Service or the Army Nursing Service Reserve. As it had been generally understood that in the military hospitals in South Africa only nurses of the Army Nursing Service Reserve were being employed, it was felt that the work of the unqualified nurses would reflect upon their more qualified sisters, and at the express desire of the President of the Army Nursing Service Reserve a letter was written by Lord Wantage, as Chairman of the Central British Red Cross Committee, to the Chairman of the Central Good Hope Red Cross Committee, suggesting that the latter committee should take such steps as they might consider expedient to make it generally known that nursing sisters employed to supplement the Army Nursing Service in South Africa were of two classes, viz.:—

(1) Those enrolled in the Nursing Service Reserve with qualifications similar to those required for the regular service; and

(2) Those employed locally for the selection of which the Central British Red Cross Committee and the Army Nursing Service Reserve were not responsible.

In reply to this letter the Chairman of the Central Good Hope Red Cross Committee wrote explaining the position of affairs. The demand for nurses was so great at one time that the military authorities in South Africa were obliged to take from time to time a proportion of partially trained nurses with a view to keeping up the supply, and to meet the probable requirements of the whole campaign. This was done by the military authorities on their own initiative. He further stated that, so far as he could learn, over 200 nurses had in this manner been engaged locally "of whom as a body nothing but good can be said." He further suggested that the Army Nursing Service Reserve should confer upon the Central Good Hope Committee autho-

### THE ARMY NURSING SERVICE RESERVE AND THE SOUTH AFRICAN WAR.—*Continued.*

rity to enrol nurses locally as nursing sisters of the Reserve, and that his committee would take care that none but properly and duly qualified persons were admitted to the privileges of this enrolment. This proposal was favourably received by the Army Nursing Service Reserve at home, and the Central Good Hope Red Cross Committee was informed that any nurses selected by the ladies appointed to represent the Reserve in South Africa would be enrolled in the Army Nursing Service Reserve on the submission of the necessary documents. In this way the general requirements of the Army Medical Service, as regards the supply of nursing sisters from amongst voluntary applications, was organised in such a way that only those fulfilling certain specified requirements as regards qualifications were selected. The general result of this has been good, and the Red Cross Committee desire to recognise the very great advantage that has been derived from organisation in time of peace of an Army Nursing Service Reserve as a channel through which selection should be made for nursing duties in military hospitals in time of war.

### Dumb Ward Pets.

BY A NURSE WHO LOVES ANIMALS.

Sometimes I like to sit quietly and bring to mind the menagerie of ward pets I have known in the course of a good many years of hospital life.

And I love to recall the pleasure some of these delightful pets have given to generations of sick people, and the happiness their mere presence has brought to a ward full of patients.

There was Jumbo—a wise old tortoise who had lived, moved, and had his happy being for ten years in a children's ward in New York. I hope he is not dead, but it is a long time since I received a bulletin as to his health and doings. His curious wanderings about the ward—his clumsy gait, his air of antiquity and wisdom caused many a sick child to forget his really sharp pain. And to have Jumbo on the bed was the highest reward the staff nurse could promise to a little patient for "star" behaviour when the surgeons came round.

### A Fox Terrier.

Who, having once known her, could forget the charms of "Nurse Judy," a fox terrier who for fifteen years was a close and loving canine friend to every little inmate of the children's ward of a London hospital. None knew better than Nurse Judy that a dog who lives in a hospital must learn to subdue her barks to a soft key. For here were little sick children whose sleep must not be disturbed even by the privileged pet of the ward. Barking was the only good thing in a doggy life that was denied her. But she cheered up under the silent ordeal, learned to express her joy by wagging her stumpy tail more than most of her kind, and was the merriest little hospital dog in the world. Another terrier, "Gypsy," has taken her place, and is already so wise a probationer that some of the nurses say they could almost trust her to take a temperature. She plays ball with convalescents, and always has a Santa Claus stocking of her own at Christmas. The only liberty she will not tolerate from her children is when they attempt to commandeer any of her own special property from the "terrier's toy-drawer."

### A Monkey.

The only time I ever saw a monkey as a ward pet was once in a little hospital in Italy. It was at Leghorn that I made Giovanni's acquaintance. Originally he had belonged to a sick Italian whose dying wish was to have his monkey with him at the last. And the good Catholic sisters didn't quite know what to do with the awkward legacy bequeathed to them by a friendless sick man. Giovanni's big eyes said as plainly as possible, "Don't send me out to face a friendless world. Isn't it enough for a monkey to lose his loving master without losing the comfortable home he has found here?" His Simian eloquence won the case. The sisters had really grown very fond of him, and the "Count," as he was popularly called, became as much of a fixture as though he had been one of the pillars of the hospital gate.

### Magpies.

Two sedate Australian magpies were the pets of a well-known hospital in Ceylon. They were popularly known as "the undertakers," and their circumspect and somewhat mournful mien almost justified a nickname which is confessedly a bit too suggestive for a hospital pet. But really they had such an uncanny way of looking at you, that you couldn't help feeling they knew the exact moment of your interment, and were quite prepared to make all the arrangements for giving you a decent funeral at "the lowest rate compatible with style and elegance."

### Goldfish.

I had forgotten the goldfish. So many hospital wards contain globes of pretty, stupid goldfish who never take the least interest in all the interesting things that are going on around them. They do not even know the difference between a surgeon and a physician. The tortoise did. He could diagnose an obvious distinction like that with his eyes shut.

### Chameleons.

I have known two chameleons who lived and died in a children's ward. They did not live long—chameleons never do in captivity—but it was good to watch the children's faces when the weird little creatures changed colour before their very eyes. A wise little boy-patient once informed the ward that it was "only conjuring like that chap did at our school treat." And ever afterwards all the new cases were told the same thing by "old hands." But it was never quite clear whether the supposed conjuring was a trick of the chameleons on their own account, or a piece of shameless jugglery on the part of sister who owned the curious couple.

### A Hedgehog and a Mongoose.

A hedgehog makes a lovely hospital pet. He amuses the patients all day, and himself half the night by hunting and killing "blackbeetles" in the kitchen. But one of the most charming of my natural history reminiscences is a mongoose who was the adored of a Calcutta hospital. He was not a ward pet. The whole hospital belonged to Rickie. You never knew where he would turn up next. Once an old native woman found him curled up in her bed. A white woman would certainly have gone into hysterics at feeling the soft, mysterious sinuous body against her limbs. But the woman of India did not mind in the least. This was one of the few Rickies I ever knew who did not sometimes show his little cruel teeth in anger. A mongoose can give a very nasty bite if he likes—and he often does like. But this creature seemed really "free from vice" of any kind, and enjoyed his happy little life of restless activity. He was for ever on the move, here one moment, gone the next, but always turning up to his meals with true masculine instinct. What a delightful book might be written on ward pets. Here am I at the end of my allotted space, and am now only on the outer fringe of my reminiscences of the charming animals I have met and known in the course of my hospital wanderings. Perhaps some day I may send a sequel to the story.

### The Red Cross Nurses of Germany.

BY A CORRESPONDENT.

The Red Cross organisation of Germany may be likened to a beneficently spreading tree, its trunk rooted in Prussia and sending out branches into all the States of the German Confederation. The branch in Baden may be taken as the type of one in full vigour, fostered and cherished as it is by the Grand Duchess and by many German ladies interested in the question of nursing. The need of having a supply of skilled nurses available in time of war was felt after the agreement arrived at by the civilised powers at the Geneva Convention to guard from injury those attending to the wounded on the battlefield. It was then that the Red Cross Society was formed, and, although owing to 30 years of peace and prosperity, the nurses belonging to it have been mainly employed in civilian hospitals, yet they are all liable to be called out on active military service. In this connection I may say that the Order of St. John of Jerusalem trains men also to help the wounded on the battlefield, and it is the fashion amongst the Prussian aristocracy to be Knights of this Order.

### The Baden Mother-House.

The Ludwig Wilhelm Home in Karlsruhe is the centre of active, loving work, training young women to become useful nurses. Candidates must be between the ages of 21 and 35, and full inquiry is made as to their fitness for the profession of nursing, not only as regards their physical but also their mental and moral qualities. If accepted, they join a preparatory course of six weeks, beginning either on February 15th or on August 15th. The authorities at Guy's Hospital are, I see, adopting this very sensible arrangement; the information gained theoretically during these weeks naturally prevents a girl being overwhelmed by her first experiences in a hospital ward. After this "preliminary canter" a candidate becomes a pupil, but she is still on probation for three months more, at the end of which time, if found suitable, she signs an agreement to serve the Baden Union for three years, or on resigning without permission to repay the cost of her maintenance, reckoned at £9 yearly.

### Where Trained.

Nurses are trained not only in the capital, but also in the large hospitals of Heidelberg, Mannheim and Pforzheim. They may belong to any Christian sect, but Jewesses are excluded; Jewish nurses have an organisation of their own in Germany. Although freed from the strict rule of Evangelical deaconesses, each nurse on signing her agreement promises to be "faithful and true to her calling, to live in peace and concord with her fellow nurses, and by her Godly and upright conduct to make as her own the motto of the Union: 'God with us.'"

### How Trained.

During the first year the pupil nurse is taught both theoretically and practically, and if possible she is given special cookery lessons. She only receives a payment of 10s. monthly, and she may leave at any time by giving 14 days' notice, but she must pay back the cost of her board. At the end of a year, if she is thought satisfactory, she becomes a "lehrschwester," or probationer. This is considered an important step in her career. She is formally received into the sisterhood; she is given a distinctive cap and a salary of £12. In another year or rather longer, if she is not considered fully trained, the probationer becomes sister and her salary gradually increases until it reaches the maximum of £24 after 12 years' service. This final reception into the Union is solemised by a service of dedication in the chapel of the Mother House. At the same time she receives a badge, a brooch showing the red Geneva Cross on a white ground, with the Union's motto to be henceforward always worn as a proof of the confidence shown in her and as a sign that she will ever prize and value her membership in the nursing sisterhood.

### Rules for Leave and Pensions.

Each sister is granted at least three weeks' annual holiday; the time may be extended on full pay if her health demands it, but otherwise pay ceases after four weeks' absence, and if away for more than six months the normal increase in her salary is suspended for a year. These rules allow a nurse to remain at home for some time, if her family circumstances require it, without severing her connection with the union. Five per cent. is deducted from the sister's salary for insurance and further payments are made by the union, so that after ten years' service, if certified unfit for duty, a nurse is entitled to a pension of £15 yearly. After that 10s. is added each year until the maximum of £20 is reached, to which she would be entitled after 20 years' service. Instead of a pension she can be received into the Mother-House, where she would be entirely maintained and nursed if ill.

### How Employed when Trained.

Once fully trained, the sisters may be put in responsible positions in hospitals, be sent out as district nurses amongst the sick poor, or be employed in private families. Seventeen rules are laid down mainly to guard the nurses' interests when sent to nurse a case, but fewer hours for rest and recreation are expected than in England. Care is taken that she gets sufficient food, for, after laying down the rule that she must not have her meals either in the sick-room or with the servants, it is ordained that they are to consist of:—Breakfast, coffee and rolls. Luncheon. Dinner, soup, meat, and vegetables, with wine or beer. Afternoon, coffee and rolls. Supper, soup, or tea and meat. Tea or coffee during the night if she is on night duty. The charge for a private nurse is 3s. 6d. a day, or 3s. if she is only required at night. A special nurse may be engaged for a case beforehand if a retaining fee of 2s. a day be paid until she is needed. The rules for district nurses are much the same as in this country, but should no other help be available they are expected not only to clean and tidy the sick-room, but also to cook and wash if there are young children in the family. They are not allowed to sit up at night except on the parish doctor's express demand, and they may never be up two nights in succession.

### Training of Housekeepers.

In the home at Karlsruhe a candidate may elect to train as a "home sister," in which case she only learns the theoretical nursing of the preliminary course, then turns aside from the path leading into a hospital ward, and takes up instead any branch of domestic economy in which she wishes to perfect herself. Thorough training is given in the kitchen, laundry and sewing-room, and every student is taught the science of house-cleaning and store-keeping. Such a special course would be of immense value to many an English nurse, who, although perhaps a distinguished ward sister, may feel hopelessly ignorant on housekeeping matters and therefore fearful of undertaking a matron's responsibilities in a small hospital, which post is often the first step towards a great one in the nursing world.

### Red Cross Matrons.

If a sister by superior intelligence and character be selected to fill the position of matron she is distinguished by a badge of black ribbon bordered with red; she changes

THE RED CROSS NURSES OF GERMANY.—*Continued.*

her grey uniform for a black one, and another special service dedicates her to her high calling. Much is expected of a matron. She has supreme control of the management of her hospital, the teaching of her nursing staff, the care of instruments, and the feeding of the patients. Not only this, but she is urged to take great interest in her young nurses, to carefully watch over their health, and by the encouragement of singing and other harmless amusements, to make the hospital as happy a home for them as possible. She has to submit quarterly reports on each pupil or probationer to the Central Committee; but when the training is completed, only half-yearly reports are sent in, and then only if there is any change to chronicle. Information is required on the following points: (1) Temperament and character; (2) intellectual development; (3) health; (4) nursing skill; (5) conduct in the ward; (6) truthfulness; (7) trustworthiness and faithfulness to duty; (8) order and cleanliness (9) how employed. A much coveted honour is a cross given by the Grand Duchess for distinguished service after ten, fifteen, or twenty years' work, made respectively of steel, silver, or gold.

### On Coronation Duty at Westminster Abbey.

BY A NURSING SISTER.

All the nursing staff had to be at their posts and take up their duties at Westminster Abbey at 7 o'clock on Coronation Day. The nursing staff comprised three superintendents, two nursing sisters of the Army Nursing Service, and two nursing sisters of the Army Nursing Service Reserve. There were four stations erected in different places about the Abbey, two in Cloister Green and one in Poets' Corner Green and one in a small room inside the Abbey. These dressing stations consisted of one or two marquees, and a sister had charge of one each. She was responsible for the control of everything connected with her marquee, and had to see that the orders of the officers in connection with patients handed over to her care were carried out. The marquees were beautifully fitted up and equipped with two beds in each marquee, also a screen, easy chair, etc., and medical comforts, and a very nice carpet with a strip of linoleum in the open used as a passage. On a table in the centre of the marquee were arranged the medical comforts close at hand in case of emergency. There was a plentiful supply of good things, such as Brand's essence, champagne, sterilised milk, cocoa, tea, brandy, and soda water, all in readiness for the occupants of the Abbey in case of faintness or fatigue. On the afternoon of Friday all the sisters who were to be on service at the Abbey were at Cloister Green to take over their duties and see their different stations, so as to be at home in their parts on Coronation Day, a kind of rehearsal which was welcome to all concerned. Each knew exactly where to go on Saturday morning at 7 A.M., and what she was to do.

The Sisters' Duties.

The care and distribution of the medical comforts was in the hands of the sisters, and each kept an expense account carefully on a manuscript sheet which was supplied to her. At the end of the day's work she handed this to the senior medical officer. Another of her duties was to keep the key of a "Field Companion," containing different kinds of drugs, which she was to give only by order of the medical officer as required. Each sister had to see that her marquee had a plentiful supply of water to start with in the morning, and to ascertain from the officer in charge how supplies of any necessaries she might require were to be obtained. The non-commissioned officers and the orderlies Royal Army Medical Corps assisted the sisters in many ways in carrying out their duties. They were engaged in ambulance work, but were ready to give extra aid if required by the sisters, and were close at hand. There was a plentiful supply of hot water which the orderlies had ready, and there was a good demand for it for various purposes, such as tea, hot-water bottles, etc. There was also a reserve marquee, as well as the others, and there was ice, and medical equipment, ice-bags, and miscellaneous equipment ready in case any of it should be necessary.

Distinguished Patients.

The sisters had the honour of receiving and ministering to some distinguished patients at the different stations, but happily nothing of a serious nature occurred to mar the pleasant anticipation of the loyal people who had assembled to witness the splendid ceremony which seemed to go on like clockwork without a hitch anywhere; and it was with much satisfaction that these distinguished patients were able after a short rest to resume their way again. Some were suffering from headache and over-fatigue, and rested on the couches provided for them in the marquees for a few hours until they felt well enough to get about again. The sisters did not leave their posts on Saturday until dismissed by the officer in charge. They remained at their posts until all the occupants of the Abbey had taken their departure, and, although they had a long day, everyone was very pleased and proud to have been able to take part in such an important, historical, and long looked-for event as the Coronation of King Edward and Queen Alexandra.

### Coronation Entertainment by the Nurses of the Royal Isle of Wight County Hospital.

BY A VISITOR.

Of all the celebrations in honour of the great event of last Saturday it would surely be difficult to find one which gave more satisfaction to all concerned than the entertainment given by the nursing staff at the Royal Isle of Wight County Hospital at Ryde. Not the least interesting part of the proceedings to the visitor, unaccustomed to hospital life, was the gathering together of the audience. This necessarily being no easy task, it began a full hour before the time fixed for the commencement of the entertainment. Those few of the patients who were able made their way unaided to the large out-patients' room, which was fitted up with a stage and gaily decorated for the occasion; but there happened to be an unusually large proportion of patients with "legs," and it required the willing help of the whole staff—nurses, maids, and porters—to get these into suitable places. As soon as this was done, the nurses retired to the "green room" to array themselves for their new rôles, and the room soon filled to overflowing with ex-patients, the patients from the adjoining convalescent home, and a very few privileged friends. All were glad to welcome the

### CORONATION ENTERTAINMENT.—*Continued.*

chairman of the hospital committee, the Rev. W. H. E. Welby, and Mrs. Welby.

The Vicar of Ryde, the Rev. J. Shearme, opened the proceedings with so much genial humour that, at the outset, performers and spectators were put into a proper frame of mind for enjoyment. All concerned, indeed, carried out their parts with the greatest spirit, from the indefatigable stage-manager (Miss Antram, the matron) down to "Buttons," who made a great success in the rôle of Mother Hubbard's dog.

The first part of the programme consisted of a series of tableaux-vivants. The picturesque costumes (all hospital-made, the work of the "artistes" themselves) aroused the greatest admiration. The Seasons first appeared, gaily apparelled; then, as a contrast, came the gruesome sight of the heads of the "late Mrs. Bluebeards" (as the chairman of the evening described those unfortunate ladies), while the arch murderer himself, as he glowered over poor Fatima, caused thrills of horror. Then "Dream Faces," in a weird and varied procession behind a window frame, passed before Sister Carr, as she reclined, seemingly asleep. The children hailed with delight their old favourites, Cinderella and her fairy godmother (Sister Yate, of the Convalescent Home), and joyfully acclaimed those monarchs of nursery fame—the King in his counting-house, counting out his money, and the Queen in her parlour, eating bread and honey. Then took place a village wedding, Sister Knight and Sister Bumpus (in Highland garb) being bride and bridegroom for the nonce. A numerous and highly picturesque group of nations, forming a phalanx round Britannia (Sister Strange), brought the series of pictures to a suitable end.

Here came the second part of the programme, the mirth-provoking waxworks, exhibited by the famous Mrs. Tarley (Nurse Palmer), with the aid of her no less notorious boy John (Sister Bateman). The audience bubbled with laughter over their jests and discomfitures, though, on the whole, the waxwork figures behaved in a remarkably life-like manner. Mother Hubbard, as became her, went to her cupboard, little Bo-peep wept for her tail-less sheep, Red Riding Hood carried a well-filled basket to her grandmother, Little Boy Blue blew his horn, the "Dirty Boy" screwed up his eyes to keep out the soap, Mary had her little lamb, Little Miss Muffet screamed at the spider that sat down beside her, Sairey Gamp made good use of her black bottle, and Jack and Jill fell down the hill with great noise and clatter—all to the great delectation of the spectators, who showed their appreciation by (considering their limitations) thunderous applause.

Intervals of waiting had been filled by songs by Nurse Palmer (the audience joining in wherever possible), pianoforte solos by Miss Taylor, and a running commentary upon the stage by the Vicar. In the course of the evening the National Anthem was sung (all duly standing except those patients with "legs"), and enthusiastic cheers were given for the newly-crowned King and Queen. Indeed, the occasion of the celebration was never allowed to drop out of sight for a moment, even the Fairy Godmother's team and Bluebeard's wives being adorned with the national tri-colour.

When the curtain fell (or rather was pulled together) for the last time, hearty cheers were given for the nurses, and then the performers emerged from the Green Room to superintend the dispersal of their grateful audience. By this time the able-bodied population of Ryde were gathering on pier and sea-front to view the illumination of the fleet, ordered by His Majesty, but nowhere could there have been more joy and satisfaction than in the hearts of the devoted staff of the Royal County Hospital, as once more they resumed their "common round."

### Everybody's Opinion.

**[Correspondence on all subjects is invited, but we cannot in any way be responsible for the opinions expressed by our correspondents. No communication can be entertained if the name and address of the correspondent are not given as a guarantee of good faith, but not necessarily for publication. All correspondents should write on one side of the paper only.]**

### PRIVATE NURSING.

"Inquirer" writes: I hear many nurses speaking about the treatment they receive from the matrons of the homes to which they belong, but how would they like this treatment? A nurse was dispatched from a home to a small-pox case in one of the large towns in South Wales, and because the case only lasted two weeks she was sent away at a moment's notice and was not allowed to take another case for two weeks. Nor was she paid the quarantine money. The matron got that as well as £8 8s., but the nurse risked her life and got nothing. When will nurses have fair play?

### A HOLIDAY QUESTION.

"A Mental Nurse" writes: I have been a nurse in a county asylum for nineteen months, and asked for my annual holiday (not having had a holiday since I have been here) to be taken in my month's notice. I was informed by the matron that because I was leaving I could not claim it, I told her I would take it, and she rejoined that I spoke as though I could do as I liked. I then saw the medical superintendent, and he informed me that as my name and date when I wished to take my holiday were not written down on a paper put up for the convenience of the nurses' annuals (not compulsory) I could not claim, but if I liked I could put the matter before the Committee of Visitors. On my saying that other nurses got their holidays granted in their month's notice, I was told that that did not concern me. I may add that I obtained good characters from both medical superintendent and chaplain before asking for my holiday. The rules are eight days annual, one day longer each year. I should like to know other people's opinion as to whether I have been treated fairly.

### TRAINING IN WORKHOUSE INFIRMARIES.

"Kathleen" writes: Will you permit me, through your columns, to inquire why the matrons of our workhouse infirmaries require three years in which to find out the capabilities of the nurses training under them? In our general hospitals, both in London and the provinces, no nurse is kept for three years' training who does not show some of the qualifications necessary to make a good nurse; but one at least of the workhouse infirmaries appears to take a different view, as the following instance will show:—A friend of mine has just completed three years' training, and was given her certificate without any apparent hesitation; great was her surprise afterwards when, applying for a vacancy on a staff of private nurses, to find she was "shut out," as the matron of her training school said she was "too nervous" for private work, although previously she had offered to obtain a vacancy for her on the private staff of one of the provincial hospitals; she has since applied for a post in one of the fever hospitals with the same result. In each case her manner and appearance were, I am told, in her favour. During her training she was placed in heavy wards and nothing ever transpired to lead her to suppose her work was not satisfactory. I think you will agree with me that if probationers do not show sufficient aptitude at the end of a year for their work they should not be retained on the staff. Necessarily the training is trying to any constitution, and if it is all to no purpose it is simply waste of time and strength to continue. In the present instance had we foreseen the result we should gladly have paid the money forfeited in the event of the agreement being broken. I have been a nurse for eight years, four of which I trained in a large general hospital in the Midlands, and I honestly confess I am glad that Fate did not lead me to a workhouse infirmary.

### Appointments.

[No charge is made for announcements under this head, and we are always glad to receive, and publish, appointments. But it is essential that in all cases the school of training should be given.]

BOLTON INFIRMARY.—Miss J. E. Such has been appointed sister. She was trained for three years at the Royal Infirmary, Derby, where she also did sister's duty for some months. She has since been nurse at the Borough Hospital, Kidderminster.

BOROUGH HOSPITAL, BOLTON.—Miss E. Weir has been appointed charge nurse. She was trained at the City of Glasgow Fever Hospital. She has since been nurse at Darlington General Hospital, and has been engaged in private nursing.

CITY HOSPITAL, WAKEFIELD.—Miss Edith Roberts has been appointed sister. She was trained at the Meath Hospital and County Dublin Infirmary, and at the Grimsby and District Hospital, and has since been staff nurse at the General Hospital, Weston-super-Mare, for one year, and at the County Hospital, Newport, Mon.

CITY ISOLATION HOSPITAL, COVENTRY.—Miss M. Ada Griffith has been appointed charge nurse. She was trained for three years at Stockport General Infirmary, and has since been charge nurse at Birkenhead Fever Hospital, staff nurse at the Hospital for Women, Soho, London, sister at the Hospital for Diseases of the Throat, Golden Square, London, and private nurse in connection with the West of England Co-operation.

JENNY LIND HOSPITAL, NORWICH.—Miss Marshall has been appointed sister. She was trained at the General Hospital, Birmingham.

JESSOPP HOSPITAL FOR WOMEN.—Mrs. Marie Elena Wolfe has been appointed sister. She was trained at the West London Hospital, Hammersmith, and at the Women's Hospital, Woolwich. She has since been midwife at Greenwich Union Infirmary, and district nurse at Brixham, Devonshire, Llanfaes, Brecon, and Bunbury near Tarporley, Cheshire.

LAMBETH INFIRMARY.—Miss May Elizabeth Brentnall has been appointed sister. She was trained at Chorlton Union Infirmary, and has since been charge nurse at Hinckley Union Infirmary for two years, and sister for nearly a year at Shoreditch Infirmary.

LIVERPOOL HOSPITAL FOR CONSUMPTION.—Miss Helen Roberts has been appointed matron. She was trained at Liverpool Royal Infirmary, and has since been sister at the Liverpool Hospital for Cancer and Skin Diseases, and sister at the Sanatorium, Kingswood, Delamere Forest.

MANCHESTER CHILDREN'S HOSPITAL, PENDLEBURY.—Miss Edith Rostance has been appointed staff nurse. She was trained for three years at the Walsall and District Hospital.

ROYAL HALIFAX INFIRMARY.—Miss Cross has been appointed sister. She was trained at the General Hospital, Nottingham, and the Hospital for Women at Liverpool. She has also been on the private staff of the latter hospital for two years.

ST. ANDREW'S HOSPITAL, NORTHAMPTON.—Miss Florence Smith has been appointed chief nurse with matron's duties. She was trained at the Glasgow Western Infirmary, and has since been deputy matron at Roxburgh District Asylum, Melrose, N.B.

STANDERTON HOSPITAL, SOUTH AFRICA.—Miss Louisa Basan has been appointed matron. She was trained at St. Bartholomew's from 1889 to 1893, and has since been charge nurse and night superintendent at Fountain Hospital, Tooting, and assistant matron at the North Western Hospital, London, for three months. In 1898 she went to the Herbert Hospital, Woolwich, and was then sent out to South Africa, retiring *pro tem.* from the Army Nursing Service in March 1901. She returned to South Africa in January 1902.

STIRLING DISTRICT ASYLUM, LARBERT, N.B.—Miss Margaret S. Gordon has been appointed assistant matron. She was trained at the Western Infirmary, Glasgow, and has for a few years past been engaged in district nursing at Thornliebank, near Glasgow.

WEST NORFOLK AND LYNN HOSPITAL.—Miss Annie Burland Todd has been appointed matron. She was trained for three years at the Birmingham Infirmary, and has since been for four years at St. Bartholomew's Hospital, London. For one year she was attached to the private nursing staff at St. Bartholomew's. She has also been home sister at Crumpsall Infirmary, matron at the Children's Hospital, Linthorpe, Middlesbrough, and temporary matron, doing holiday duty, at the Birmingham and Midland Eye Hospital.

WOODLANDS CONVALESCENT HOME, RAWDON.—Miss Alice Riley has been appointed matron. She was trained at the London Hospital, and has been matron at the Skipton and District Hospital since its opening in 1899.

### Presentations.

SKIPTON DISTRICT NURSING ASSOCIATION.—Miss Welburn who is leaving Skipton in order to take up the post of nurse at Woodlands Convalescent Home, Rawdon, has been presented with a valuable timepiece by the members of the committee of the Skipton District Nursing Association, as a mark of their appreciation of her services.

### TRAVEL NOTES AND QUERIES.

CONVENT NEAR AUGSBURG (Deaconess).—Your letter is so illegibly written that I cannot read the name of the place about which you inquire. Write again, please, very distinctly. I gather that it is a religious colony, but I can find no such place in any register. Augsburg is in South Germany, but the address reads as if the convent is in a valley—thal means valley—and yet it is Augsburg, which is a large city. Kindly give me more clear information. The route to Augsburg is viâ Harwich, the Hook, and Cologne. Cost, second return, about £4 10s. The spring is generally the pleasanter season on the Continent, but autumn would be quite as suitable.

CYCLING TO BOURNEMOUTH (B. A.).—The information I wanted for you is to hand, but the route is by Winchester. Why not go by Salisbury and return by Winchester. My friend went by Hammersmith, Staines, Egham, Basingstoke, Winchester, Romsey, and Ringwood. If you wish to stay a night in Winchester, I think you would like the Black Swan Hotel. Be sure to visit St. Cross. The Cathedral is much finer inside than Salisbury, but lacks the graceful exterior and the unique close.

FOREIGN HOTELS (K. H.).—We do not reply by letter unless we receive 2s. 6d. for our Convalescent Home. Our correspondence being large it is difficult to remember to what place you allude, but from your remarks I think it must be Dieppe. It would be invidious to give you the distinctive information you ask for, and hardly fair to the hotel-keepers; moreover, I really believe there is but little difference between one and another. From a tolerably wide experience I can speak gratefully of the kindness and consideration usually met with. I never believe grumblers; if they encounter unpleasantness it is usually their own fault. At the terms you offered (if I recollect aright) the hotels I named would not be first class and you must not expect luxury; but cleanliness, good food, and obliging kindness, I think you will find without doubt.

### Echoes from the Outside World.

### THE CORONATION.

### The King's Message to his People.

Prior to the Coronation the King issued a message to his people which, though sent out through the medium of the Home Secretary, took the form of an autograph letter, which will be historical. It was as follows: "To My People,—On the eve of my Coronation, an event which I look upon as one of the most solemn and important of my life, I am anxious to express to my people at home, and in the Colonies, and in India, my heartfelt appreciation of the deep sympathy which they have manifested towards me during the time that my life was in such imminent danger. The postponement of the ceremony owing to my illness caused, I fear, much inconvenience and trouble to all those who intended to celebrate it; but their disappointment was borne by them with admirable patience and temper. The prayers of my people for my recovery were heard; and I now offer up my deepest gratitude to Divine Providence for having preserved my life and given me strength to fulfil the important duties which devolve upon me as the Sovereign of this great Empire. (Signed) Edward R. and I., Buckingham Palace, August 8th, 1902."

### The Processions.

When dawn broke over London on Saturday morning, heralded as it was with the heavy booming of cannons, it revealed a few groups of enthusiastic sightseers who had taken up their positions over night. The first section of the Royal procession arrived at the Abbey soon after half-past 10, and a few minutes later the Prince and Princess of Wales were driven up. Exactly at 11 o'clock the firing of the guns announced that the King had left Buckingham Palace. The watermen and the King's Bargemaster appeared first, and then after the members of the Royal Household the Indian potentates on horseback, in their splendid uniforms, and accompanied by their aides-de-camp. The heroes of the South African war were greeted with rapturous cheers by the multitude, and then after the Colonial and Indian Cavalry the State coach, with its eight cream-coloured ponies came into sight. The King was richly robed, wore a Royal cap of crimson, shaped like a coronet, and looked well and smiling, appearing well pleased with the enthusiastic reception given to him and the Queen.

### The Ceremony.

The procession inside the Abbey started with the clergy, followed by five pursuivants and the representative officers of the Order of Knighthood; the four knights of the Order of the Garter appointed to hold the canopy for the King's anointing; the Prime Minister and other ministers of State, and then the Archbishops of Canterbury and York. The second portion was devoted to the Queen's regalia and the Queen in her Royal robes, supported on each side by a bishop, and her train borne by the Mistress of the Robes and eight gentlemen. As the Queen appeared, the privileged Westminster boys greeted her with shouts of "Vivat Regina, Vivat Regina Alexandra." After she had been conducted to her seat, and a few minutes had elapsed, the King's regalia followed, and then the King himself appeared in his crimson robe of state, supported by two bishops, his train borne by eight gentlemen and the Master of the Robes. He also was greeted by the Westminster scholars, and then the Archbishop presented him to the people assembled as "King Edward, the undoubted King of this realm," and asked them if they were willing to come and do homage to him. In reply all the people shouted "God Save King Edward," and the trumpets blared. After the Communion Service had proceeded as far as the Nicene Creed, a number of questions were put to the King by the Archbishop, all of which he answered in loud, clear, and steady tones. Finally he knelt down, laid his right hand upon the Bible, and said, "The things which I have herebefore promised I will perform and keep. So help me, God." Then he kissed the book, and signed the oath, pen and ink being borne on a silver standish. During the singing of the anthem the King was relieved of his cap of State and crimson robe, conducted to his chair, and whilst the canopy was held over him the Dean of Westminster poured the holy oil into the spoon, and dipping in his fingers anointed the King in the form of a cross on head and breast, and on the palms of his hands. His Majesty was next arrayed in a pall of cloth of gold, his heels were touched with the spurs, and a sword was girt about him. This sword he offered on the altar, but it was redeemed by a peer for a hundred shillings, and it was then carried naked before the King during the rest of the solemnity. The Sovereign was also given the orb, with the cross, the ring, the glove, and two sceptres, and, last of all, the crown was placed upon his head. In a moment the deep silence of the building was broken by a heart-thrilling cry from every corner of the building, "God save the King!" When the cries had subsided the Archbishop admonished the King, and then, with the help of the Archbishop of York, other bishops, and the peers, "lifted" him into his throne, and the clergy, princes, and peers paid homage to him. This was done by touching the crown on His Majesty's head and kissing His Majesty's left cheek. The Prince of Wales also rendered homage in the same way, and the King, in response, shook his son heartily by the hand and kissed him on both cheeks.

Next came the ceremony of crowning the Queen Consort, the office being performed by the Archbishop of York, who poured the oil into the spoon and thence on to the Queen's head. Her Majesty was presented with a ring, and then the Archbishop took the crown from off the altar and set it reverently on her head. At the same moment all the peeresses took in both hands the coronets which had been hitherto laying in their laps, and slowly raised them on to their heads. This simultaneous action, which resulted in a sudden blaze of jewels, was singularly impressive. After the Archbishop had given the sceptre and the ivory rod, the Queen left the Faldstool, and as she passed the King on his throne, she made a deep obeisance. As a conclusion to the service both the King and Queen laid aside their crowns and partook of the Holy Communion.

### The Return to Buckingham Palace.

Although their Majesties had been timed to leave the Abbey at one o'clock, it was past two before the King mounted, unassisted, the three steps of his coach in which the Queen was already seated. The long waiting had made the people in the streets fearful that something untoward had happened; the relief to their feelings when they saw the coach returning with the King, looking smiling and well, if a little tired, lent to their cheers an added note of thankfulness, and the enthusiasm was such as even Londoners have seldom seen. When the King and Queen had entered Buckingham Palace, King Edward VII., emerged, crowned, on to the balcony, and inviting the Queen to come out beside him, they stood there for some minutes, to the delight of the onlookers.

### The King's Gift to his People.

The King, whose health is now considered so satisfactory that no further bulletins will be issued, has signalised his Coronation by presenting Osborne House to the nation. In a letter to Mr. Balfour His Majesty, after referring to the fact that Osborne under the will of the late Queen is his private estate, explains that for various reasons he feels that he will be unable to make adequate use of the house as a Royal residence. The palace is therefore, with the exception of the apartments sacred through the personal use of Queen Victoria, to be always accessible to his people, and he hopes that it may be converted into a convalescent home for officers of the Army and Navy.

### For Reading to the Sick.

"LEARN OF ME . . . AND YE SHALL FIND REST UNTO YOUR SOULS."—*St. Matt.* xi. 29.

Oft in Life's stillest shade reclining,
In Desolation unrepining,
Without a hope on earth to find
A mirror in an answering mind,
Meek souls there are, who little dream
Their daily strife an Angel's theme,
Or that the rod they take so calm
Shall prove in Heaven a martyr's palm.

*Keble.*

---

To know Thee is on earth all blessedness,
And is to be at peace with God and man!
Nor need we wings of the transporting dove
To bear our restless souls to be with Thee
In th' Heaven of Heavens; for Thou art with us here;
Love shall give dovelike wings of prayer and alms
To bear the soul to Thee, her place of rest.
Meanwhile, 'mid stormy tumults of the world,
The Ark of Thine own presence gathers home
Thy children, and protects them o'er the wave.

*Isaac Williams.*

---

The beloved disciple St. John made his thanksgiving leaning on Jesus's Breast, absorbed in Him he loved, in perfect, unquestioning rest, although on the eve of great troubles such as he had never yet known. So if we possess Jesus, if our heart is one with His Heart, we may lie upon His Breast, not fearing sorrow, temptation, care, trial, pain, not even fearing the assaults of sin; since He bears us and all our troubles, He can heal, sustain, soothe, and strengthen us. There is rest and strength in the thought that He watches over the battle, and that one need be afraid of nothing. There is no stay so strong as an unreserved abandonment of self into God's Hands. We risk nothing, need fear nothing, in so doing. Friends in whom we have trusted may fail us; coldness and neglect or distance may loosen ties which once were strong; but He can never fall short of His promises. Give all to Him; keep back nothing; and He will give back such an abundant overflow of love and blessing, though that very love may at times seem to deal hardly with its object for its greater good.

We must strive never to lose faith in the efficacy of prayer, even when we do not see the fulfilment; we must rest lovingly in our Father's Hands, Who knows to the full all we desire, and how best it may be accomplished. We see such a little way, and he looks through all time and eternity for us. Let us leave it all to Him. "What I do thou knowest not now, but thou shalt know hereafter."

*Sidney Lear.*

---

How to all the sick and tearful
Help was ever gladly shown;
How He sought the poor and fearful,
Call'd them brothers and His own.

How no contrite soul e'er sought Him
And was bidden to depart,
How with gentle words He taught him,
Took the death from out his heart.

Let me kneel, my Lord, before Thee,
Let my heart in tears o'erflow,
Melted by Thy Love adore Thee,
Blest in Thee 'mid joy or woe!

*L. Hensel.*

### Notes and Queries.

**The Editor is always willing to answer in this column, without any fee, all reasonable questions, as soon as possible.**

**But the following rules must be carefully observed:—**

**Every communication must be accompanied by the name and address of the writer.**

**2. The question must always bear upon nursing, directly or indirectly.**

**If an answer is required by letter a fee of half-a-crown must be enclosed with the note containing the inquiry, and we cannot undertake to forward letters addressed to correspondents making inquiries. It is therefore requested that our readers will not enclose either a stamp or a stamped envelope.**

---

*Remedial Treatment.*

(141) Will you kindly tell me at what institution (public or private) I could get lessons in "Remedial Treatment and Physical Culture," also the names of the best books treating the subject?—*Trained Nurse.*

If "Trained Nurse" means remedial treatment of disease, surely the "lessons" which she inquires about must include the whole study of medicine! As for the term "physical culture," that is a phrase which has come into use of recent years, and to which many meanings are attached. Calisthenics, gymnastics, and various "exercises," to some of which the more astute teachers have attached their own names, are often included, but probably no two "professors" would quite agree as to the exact meaning of the term.

*Rheumatism.*

(142) Could you kindly recommend any place for lady suffering from rheumatism, who wishes to take the baths. She can only pay a small fee, and the place must be within easy reach?—*District Nurse.*

We cannot prescribe. "Rheumatism" is a condition which presents many varieties. Ladies vary even more. If the question had merely been what baths are useful in rheumatism, we could have given a long list, but such a list would be no help in deciding about an individual case. Consult a doctor.

*South Africa.*

(143) Can you tell me if there are any asylums in Capetown where probationers are trained? I am over age for general training.—*F. M. R. and Nurse D.*

You had better write in the first place to Dr. W. J. Dodds, Inspector of Asylums, Valkenberg Hospital for the Insane, Mowbray, Capetown.

Are there any good openings in South Africa for nurses? I have had twelve months' private training and hold the Medico-Psychological Society's and the L.O.S. certificates. Will you kindly tell me the best way to secure an appointment before going?—*Anxious.*

See replies to *J. C.*, *F. M. R.*, and *Nurse D.* You might also write to the Lady Superintendent, the Victoria Nurses' Institute, Capetown.

Will you kindly tell me the addresses of the Nurses' Co-operative and also of some of the mining hospita's at Johannesburg? I have a three years' certificate in general nursing, fever training, and a knowledge of midwifery.—*N. N.*

Write to the Superintendent Nurse, the Co-operation Residential Club, Johannesburg.

I am a certificated maternity nurse and have a desire to go to South Africa. Will you kindly tell me if there are any institutions which send out nurses, and if so what are the terms?—*R. B.*

No; nurses going to Africa on their own account must pay for themselves. See replies to other nurses under this heading.

*Feeble-minded.*

(144) I have been reading an article in The Hospital, and think that it would be a good thing if I could get a little nephew of mine into one of the homes for the feeble minded. He is bright enough in some respects, but the boys tease him at school.—*Lucy.*

Apply to the Secretary, The National Association for Promoting the Welfare of the Feeble-minded, 53 Victoria Street, S.W.

**Standard Nursing Manuals.**

"The Nursing Profession: How and Where to Train." 2s. net; post free 2s. 4d.

"Art of Massage." (Second Edition.) 6s.

"Elementary Physiology for Nurses." 2s.

"Elementary Anatomy and Surgery for Nurses." 2s. 6d.

"Practical Handbook of Midwifery." 6s.

"Surgical Ward Work and Nursing." Revised Edition. 3s. 6d. net; post free 3s. 10d.

"Mental Nursing." 1s.

"Art of Feeding the Invalid." 1s. 6d.

All these are published by the Scientific Press, Ltd, and may be obtained through any bookseller or direct from the publisher, 28 and 29 Southampton Street, London, W.C.

### Travel Notes.

By Our Travelling Correspondent.

### CVI.—WHAT WE CAN SEE FOR FIVE POUNDS.

### In the Valley of the Meuse and the Lesse.

I do not think you could do better than Dinant or Anseremme on the Meuse if you have a week's holiday and do not want to spend more than £5. Possibly, Anseremme might be a trifle cheaper, and it is most beautifully situated at the junction of the Lesse and the Meuse, but it is not quite so conveniently placed for trains, steamer, etc. It is about one and a quarter mile from Dinant, and if you are fair walkers or cyclists, that disadvantage would be insignificant.

### The Cost of the Journey.

Second-class return to Namur *viâ* Harwich and Antwerp is £1 14s. 2d., thence by steamer costing two francs or under. It is desirable to make this last part of the trip by steamer, if possible, because the beauty of the river banks is almost entirely lost by taking the train, but in a dry summer the water is often too low to admit of it. If you are cyclists go by road from Namur to Dinant.

### Attractions of this Trip.

The great charm of the Ardennes district is the country itself, its green and fertile valleys, its rich wooded hillsides, its winding rivers and nestling villages. There are very few architectural features, except here and there an interesting castle or manor house, more or less in decay, so if your tastes incline to old cities and buildings I should counsel you to turn your attention to some of the places I have been describing during the past few weeks. But for good, or even moderate walkers who desire absolutely pure air and fresh green scenery, the Ardennes will exactly meet your requirements.

### La Roche.

Before speaking of Dinant and its immediate surroundings I should like to mention the little town of La Roche. It is, I believe, a very good fishing place, which makes it attractive to many, and the country is beautiful; but it is not such a good centre as Dinant for exploring. It lies on the river Ourthe and the walks are really charming; for a very quiet week it would be ideal, but there is nothing like the variety to be found in and round Dinant. There is now a light railway from Melreux, and it is quite accessible, but at my first visit I remember I was obliged to share the only vehicle which Melreux provided with three amiable but rather rowdy young artists, who were politeness itself to me but indulged in a little horseplay among themselves, and we rattled up to the respectable inn at La Roche to the strains of some Bacchanalian ditty which corresponds somewhat to our English "We won't go home till morning."

### Dinant and its Immediate Surroundings.

I shall not enter upon the subject of hotel expenses, as it takes up so much space, but shall be happy to help you to make a choice if you settle to go there. The town consists of one very long street and a few branching from it. This principal street has some not very striking shops, which are largely filled with what are called *couques de Dinant*, a flat, rich and unpleasant kind of cake partaking of the nature of gingerbread. Another of the town's specialities is copper ware, and a sprinkling of brass pots and pans.

The great charm of Dinant itself is the river, with its picturesque reflections and its splendid church, whose bulbous spire stands out in relief against the natural rock and strong fortifications climbing up behind. Round the church's feet are grouped houses, which are the delight of the artist, with their roofs, originally red, now toned to every charming variety of tint, with arcaded lower stories, and an ever-varying throng of Flemings moving up and down beside the Musee water. I believe I ought not to call them Flemings, for they pride themselves on being distinctive, and upon belonging to the Walloon country. The church stands in the Grande Place and is particularly interesting. It is thirteenth century work and very harmonious.

Steps ascend to the citadel behind the church but it is not worth while to pay 50 centimes to visit the interior and the view is just as good outside, but from there you can sharply descend to a beautiful rocky little valley called the Fonds de Leffe where there are numerous water mills and the scenery is very pretty.

### Excursions in the Neighbourhood.

There is a beautiful excursion up the valley of the Lesse, which is a wild and singular formation, with caverns and much thick undergrowth; you turn up by Anseremme, but do not cross the bridge to the farm; keep on up the valley of the Lesse till you come to a mill, where you cross the ferry to the château of Walzin, strikingly placed on a pinnacle of rock and reflected in the sullen waters below; it is rather a gloomy spot, but undeniably most lovely. From there you can go to Chaleux and return by boat if you will.

I think you would enjoy one day in Namur, immortalised as the scene of Uncle Toby's military exploits; you can go up very easily by steamer or train, and it is a city of interest from its tremendous fortifications.

The Cathedral is really hardly worth a visit, and only dates from 1751; the same may be said of the Church of St. Loup, though it is more than a hundred years older. There is a remembrance of the siege by Louis XIV's troops, in a large hole of the ceiling, made by a cannon-ball. The belfry standing in the Grande Place is of the sixteenth century, the Hôtel de Ville is modern and without interest, but be sure to see the Ancienne Boucherie, now a museum, and the Hospice d'Harscamp, and the old stone bridge to the suburb of Jambes.

A short walk from Dinant is Poilvache, picturesque and striking, once a strong fortress but destroyed in the sixteenth century, and now only a ruin.

Nearer to the town is Bouvignes, once a considerable place and a formidable rival to Dinant, but now fallen from its high estate and given over to the domination of the domestic porker—at least pigs seem to me a striking feature of the place.

High overhead stands the tower of Crèvecœur, celebrated for its heroic defence in 1554 to the arms of France and the tragic death of the only three women left alive, who threw themselves into the river rather than fall into the hands of the besiegers. Next week I hope to tell you of some more excursions in the neighbourhood, notably that to Veve Celles, which is almost the most interesting point within reach.

### To Nurses.

We invite contributions from any of our readers, and shall be glad to pay for "Notes on News from the Nursing World," or for articles describing nursing experiences, or dealing with any nursing question from an original point of view. The minimum payment for contributions is 5s., but we welcome interesting contributions of a column, or a page, in length. It may be added that notices of appointments, entertainments, presentations, and deaths are not paid for, but that we are always glad to receive them. All rejected manuscripts are returned in due course, and all payments for manuscripts used are made as early as possible after the beginning of each quarter.

